# Stem cell niches and other factors that influence the sensitivity of bone marrow to radiation-induced bone cancer and leukaemia in children and adults

**DOI:** 10.3109/09553002.2010.537430

**Published:** 2011-01-04

**Authors:** Richard B Richardson

**Affiliations:** Radiological Protection Research and Instrumentation Branch, Atomic Energy of Canada Limited, Chalk River Laboratories, Chalk River, Ontario, Canada

**Keywords:** cancer predisposition, bone, stem cells, radiation-induced tumours

## Abstract

*Purpose:* This paper reviews and reassesses the internationally accepted niches or ‘targets’ in bone marrow that are sensitive to the induction of leukaemia and primary bone cancer by radiation.

*Conclusions:* The hypoxic conditions of the 10 μm thick endosteal/osteoblastic niche where preleukemic stem cells and hematopoietic stem cells (HSC) reside provides a radioprotective microenvironment that is 2-to 3-fold less radiosensitive than vascular niches. This supports partitioning the whole marrow target between the low haematological cancer risk of irradiating HSC in the endosteum and the vascular niches within central marrow. There is a greater risk of induced bone cancer when irradiating a 50 μm thick peripheral marrow adjacent to the remodelling/reforming portion of the trabecular bone surface, rather than marrow next to the quiescent bone surface. This choice of partitioned bone cancer target is substantiated by the greater radiosensitivity of: (i) Bone with high remodelling rates, (ii) the young, (iii) individuals with hypermetabolic benign diseases of bone, and (iv) the epidemiology of alpha-emitting exposures. Evidence is given to show that the absence of excess bone-cancer in atomic-bomb survivors may be partially related to the extremely low prevalence among Japanese of Paget's disease of bone. Radiation-induced fibrosis and the wound healing response may be implicated in not only radiogenic bone cancers but also leukaemia. A novel biological mechanism for adaptive response, and possibility of dynamic targets, is advocated whereby stem cells migrate from vascular niches to stress-mitigated, hypoxic niches.

## Introduction

To assess the potential risk of radiation-induced cancer, there is a need to calculate the absorbed dose to radiosensitive tissues or ‘targets’ and to compare this dose with the results of epidemiological studies. Leukaemia and bone cancer contribute to the total risk of cancer and hereditary disease estimated by the committed effective dose as recommended in Publication 103 of the International Commission on Radiological Protection (ICRP 103 2007). The longtime ICRP target tissue for radiogenic bone cancer is the 10 μm thick “endosteal layer” (ICRP 30 1979/81) adjacent to trabecular and cortical bone surfaces (Table I). This target was based on quiescent osteoblasts/bone lining cells that are no longer considered as possessing substantial carcinogenic potential (ICRP 11 1968). In 2007, the ICRP considered changing the thickness of the primary bone cancer target adjacent to bone surface from 10–50 μm (Bolch et al. 2007): This new thicker target will be referred to as “peripheral marrow”. Bone cancer risk is assessed from the dose to peripheral marrow found in trabecular bone cavities and medullary (long bone) cavities in cortical bone, now excluding cortical Haversian canals. The whole red marrow including the endosteum continues to be the ICRP's target tissue for calculating dose and risk of radiation-induced leukaemia.

**Table I:** Marrow niche characteristics of normal and cancer stem cells

	Endosteal/osteoblastic niche	Vascular/endothelial niche	References
Location in marrow	10 μm layer next to bone surfaces	Hematopoietic tissue	Lord (1990), Kiel et al. (2005)
Normal and cancer stem cell replication	Mainly self-renewal of stem cells	Mainly asymmetric division (produce differentiated progeny)	Arai and Suda (2007), Eliasson and Jönsson (2010)
Normal stem cell activity	Quiescent	Rapid blood cell expansion	Moore and Lemischka (2006), Nagasawa (2006)
Cancer stem cell activity	Quiescent	Rapid primary/metastatic tumour spread	Sipkins et al. (2005), Ishikawa et al. (2007), Medinger and Mross (2010)
O_2_ pressure (% atmospheric)	0.1–1%	∼7%	Parmar et al. (2007), Harrison (2002)
O_2_ enhancement ratio (OER, low-LET)	1.1–1.4	2.5	Thoday and Read (1949), Richardson (2008)

The historical emphasis of the ICRP's dosimetry has been primarily for adults. This paper reviews skeletal targets and proposes new ones suitable for all ages of human development. Richardson (2009a) assessed the risk of excess fatal cancers from internal radionuclide exposures as the product of three factors: the radiation hazard (excess mortality Sv^−1^), exogenous exposure (Bq) and internal exposure dose coefficient (Sv Bq^−1^). The National Research Council publication Report V on the Biological Effects of Ionising Radiations, i.e., BEIR V (1990), estimated children were around 10 times more vulnerable per unit dose (Sv^−1^) to fatal, radiation-induced solid cancers than middle-aged adults: leukaemia exhibited less variation with age.

Children take in smaller amounts of contaminated air and food than adults. However, in smaller bodies, radioactivity is more concentrated, turned over more rapidly due to a higher metabolism and uptake in organs elevated due to growth. The selection of appropriate radiation targets for children that have been internally exposed to bone-seeking radionuclides is important, as the infant:adult ratio of the total fatal cancer risk per unit inhaled activity (Bq^−1^) is around 10:1 (Richardson 2009a). Similarly, the infant:adult risk per Bq ingested activity is ∼100:1. The ICRP in 1989 extended radiation dosimetry from adults to children and infants, and later added the embryo/foetus (ICRP 56 1989, ICRP 88 2001).

The ICRP's skeletal targets, developed for adults, were recommended for assessing the radiation risk to non-adults. However, unlike some soft tissue organs, not only are the dimensions of the skeleton changed by growth in childhood but there are also profound age-related changes in skeletal physiology and morphology.

Since 1979, when the endosteum and whole red marrow were chosen as ICRP targets, there have been significant advances in the understanding of radiation health effects and the biology of stem cells, cancer stem cells, fibrosis, inflammation and carcinogenesis. This paper was written to meet the challenge set in 2007 by the ICRP Task Groups on Internal Dosimetry (INDOS) and Dose Calculation (DOCAL) to provide supporting biological and epidemiological evidence for proposed partitioned bone cancer and leukaemia targets. Nie and Richardson (2009) modeled the dose to stem/progenitor cells adjacent to the trabecular bone remodelling compartment and found this site to be a more likely candidate target for radiation-induced bone cancers, especially in childhood, than the ICRP target (i.e., the peripheral marrow adjacent to quiescent bone surfaces). This new target is based on the risk of bone cancer being higher when bone turnover is elevated: this is the case in the young rather than the old, in trabecular rather than cortical bone, and in association with hyper-metabolic pathology such as Paget's disease (osteitis deformans or osteodystrophia deformans).

**Figure 1:**
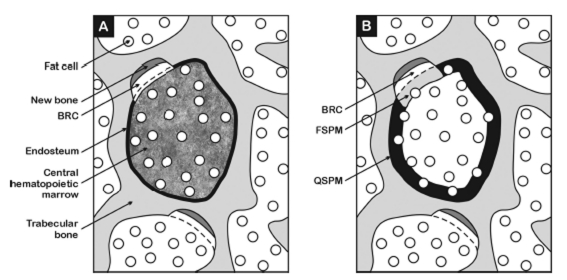
Schematic diagrams (not to scale) depict trabecular marrow cavities that in reality are usually interconnecting. Sub-figure (A) names the anatomical parts of a marrow cavity and shows the partitioned hematopoietic targets for leukemia, consisting of the endosteum and central hematopoietic marrow and (B) shows the partitioned peripheral marrow targets for bone cancer consisting of quiescent surface peripheral marrow (QSPM) and forming surface peripheral marrow (FSPM): the latter target could include the bone remodelling compartment (BRC).

The following dosimetry targets for ‘marrow neoplasms’ (i.e., bone cancer and leukaemia) are proposed as better representatives of radiation-induced risk to the third trimester foetus and at all post-natal ages (Figure 1):

- Leukaemia ‘partitioned hematopoietic targets’ – instead of the ICRP's whole red marrow, trabecular hematopoietic tissue is partitioned into two weighted parts: The endosteum/endosteal niche (reduced weighting due to hypoxia) and the remainder central cavity marrow.
- Bone cancer ‘partitioned peripheral marrow targets’ – the ICRP's whole peripheral marrow is partitioned into two weighted parts: First, peripheral marrow associated with quiescent bone surfaces, and second, peripheral marrow associated with forming bone surfaces.
- Both partitioned targets exclude marrow fat cells that have low carcinogenic potential. The dose associated with skeletal growth, remodelling and forming surfaces can be weighted to represent the greater cancer risk to children.

The contemporary knowledge on stem cells, including their niches and oxygen levels, is reviewed to determine the choice of marrow targets and their radiosensitivity, especially taking infants and children into account. It would be ideal if the calculated radiation dose to targets always predicted risk, but there are factors other than dose – such as cell proliferation, genomic instability and the prevalence of cell killing or pre-existing lesions – that affect the risk of spontaneous and radiogenic neoplasms in different populations (Streffer 2009). The influence of pre-existing lesions on the occurrence of adult radiogenic bone cancer was formulated a decade ago (Gössner 1999). Since that time, the knowledge of biological mechanisms of malignancy transformation has advanced and may further explain the development of radiation-induced neoplasms. Finally, existing and proposed marrow targets both have static locations; but in future dynamic targets could be an option as stem cells migrate in some circumstances.

## Skeletal cancer targets lie primarily in trabecular bone marrow

### (1) Spontaneous and radiation-induced leukaemia

Leukaemia is the most common childhood cancer (Table II), representing ∼31% of cancer cases in US children under 15 years of age (Smith et al. 1999). In US adults over 65 years old, leukaemia has ∼12 times higher incidence than in children, but makes up only about one-fortieth of all cancers reported (Ries et al. 2007). Acute myeloid leukaemia (AML), chronic myeloid leukaemia (CML) and chronic lymphocytic leukaemia (CLL) are primarily cancers of old age, whereas acute lymphocytic leukaemia (ALL) is particularly common in childhood. Most leukaemias originate from precursor cells in bone marrow (including B-cell lymphocytic leukaemias), a notable exception being thymic T-cell ALL which represents about 15–20% (mostly in children) of cases of ALL in Western countries. An advisory group of the National Radiological Protection Board (NRPB 2003) tasked with estimating the incidence of different types of cancers for the UK population reported that myeloid leukaemias made up just over a third of spontaneous leukaemias, whereas radiation-induced myeloid leukaemias, especially AML, were over-represented compared to their lymphoid counterparts (Table III). Thorotrast-administered patients irradiated with high-linear-energy-transfer (high-LET) ^232^Th alpha-radiation exhibit even greater domination by AML, ∼4/5ths of the leukaemia cases. An excess risk of leukaemia is also associated with the higher doses prescribed in radiotherapy for cancer (Boice et al. 1987, Curtis et al. 1994) and X-ray treatment of ankylosing spondylitis (Weiss et al. 1995). Thorotrast, radiotherapy and atomic-bomb survivor studies all reported no radiation-induced CLL (Preston et al. 1994). However, CLL's rarity among Asian populations, prolonged latency (perhaps 20 years compared with ∼5 years for other leukeamias), and misdiagnosis may have erroneously designated CLL as a non-radiogenic form of cancer (Richardson et al. 2005).

**Table II:** Characteristics of different types of leukaemia, namely their relative incidence levels of spontaneous occurrence at different stages of human development and the stem or progenitor cells from which the leukaemic types originate (Ries et al. 2007). Note that the Philadelphia chromosome is not only found in children with ALL but also found in adults with ALL, and occasionally those with AML (Jamieson et al. 2004)^a^

	Acute myeloid leukaemia (AML)	Chronic myeloid leukaemia (CML)	Acute lymphocytic leukaemia (ALL)	Chronic lymphocytic leukaemia (CLL)
Spontaneous incidence	Most common acute leukaemia in adults	Occurs in middle aged and elderly adults	Majority of childhood leukaemia	Rare under 45 years of age
Hematopoietic stem cell (HSC) and its leukaemic progenitors.	HSC, myeloid progenitors^a^	HSC, granulocyte-macrophage progenitors.	HSC, lymphoid progenitors.	HSC, lymphoid progenitors.
		Usually Philadelphia chromosome translocation present^a^	Majority are of B cell lineage^a^	Great majority are of B cell lineage

**Table III:** Comparison of the percentage incidence rates for spontaneous and radiation-induced leukaemia (assuming 0% radiation-induced CLL). The spontaneous incidence rates are those of England and Wales from NRPB (2003)^a^ as given in their Table 1.1. The low-LET incidence rates are those of the UK population, also from NRPB (2003)^a^ as evaluated in their Table 4.3 based on the BEIR V (1990) risk model. The high-LET incidence rates are those of Danish Thorotrast patients (Visfeldt and Andersson 1995)^b^

	AML	CML	ALL	CLL
Spontaneous^a^	27%	9%	9%	55%
Low-LET radiation^a^	53%	22%	25%	0%
Thorotrast (high-LET)^b^	80%	15%	5%	0%

AML, Acute myeloid leukaemia; CML, Chronic myeloid leukaemia; ALL, Acute lymphocytic leukaemia; CLL, Chronic lymphocytic leukaemia.

The excess relative risk of leukaemia in children or adults exposed to the radiation dose from atomic bombs dominated by low-LET high-energy gamma rays is more than other cancers (Preston et al. 1994). The evidence for lymphoma and multiple myeloma in atomic-bomb survivors is weak or non-existent. Brenner et al. (2003) found significant excesses of leukaemia in children reported for different public and occupational populations exposed to ionising radiation doses as low as a few mSv. The Oxford survey is the largest case-controlled study of childhood cancers associated with obstetric X-rays, mean dose 10–20 mGy (Bithell and Stewart 1975). This study found, in common with other smaller case-control studies, a proportional increase in risk of ∼40% for childhood cancer (about half being leukaemia) associated with prenatal irradiation (Doll and Wakeford 1997). However, *cohort* studies of medical or atom bomb in-utero irradiation provides limited evidence of increased risk of cancer (ICRP 90 2003).

### (2) Spontaneous and radiation-induced bone cancers

Sarcomas of the bone and cartilage are relatively rare comprising about 0.2% of all malignancies in the total US population (but a higher proportion of childhood cancers, ∼6%) – an incidence rate similar to other developed countries (Gurney et al. 1999, Ghadirian et al. 2001, Ries et al. 2007). Chrondrosarcomas, derived from a mesenchymal cartilage progenitor, are the spontaneous bone cancers most frequent in adults; however, they occur about five times less commonly as a fraction of radiation-induced bone cancers (Table V). Osteosarcomas, an osteoblastic neoplasm, are the most common form of spontaneous and radiation-induced bone cancer in a population, and are particularly prevalent in children (Tables IV and V). A risk factor for osteosarcoma is the rapid bone growth in adolescence. The intramedullary form of osteosarcoma is most common (∼75%), the periosteum form makes up 4–10% of cases, while intracortical osteosarcoma is rare (<1%) (Murphey et al. 1997). The remainder of osteosarcomas is more defined by histopathology than their location in bone.

**Table IV:** Characteristics of different types of spontaneous and radiation-induced bone tumours (Ewing's sarcoma is not associated with radiation^a^) including their relative incidence levels of spontaneous occurrence at different stages of human development, the stem or progenitor cells from which the bone tumour types originate and their location in the skeleton (Gurney et al. 1999).

	Osteosarcoma	Chondrosarcoma	Fibrosarcoma	Ewing's sarcoma^a^
Spontaneous childhood incidence	Most common	Rare	Almost unknown	2^nd^ most common
Spontaneous aged adult incidence	2^nd^ most common	Most common	Rare	Rare
Mesenchymal stem cell (MSC) and its progenitors.	MSC, osteoblastic progenitors	MSC, cartilage progenitors	MSC, stromal progenitors	MSC neural crest origin
Usual skeletal location	Metaphysis of long bones	Pelvis, leg bone and arm bone.	Metaphysis or diaphysis of long bones	Extremities and central axis
Usual bone type	Intramedullary and periosteal (Rare: intracortical)	Intramedullary	Intramedullary and periosteal	Intramedullary and periosteal

**Table V:** Comparison of the percentage bone tumour incidence rates that occur spontaneously or are radiation-induced, including malignant fibrous histiocytoma (MFH). Other types of spontaneous malignant bone tumours than are named here make up 14% of tumour types examined in ‘all population’ but only 3% of those aged 0–20 y (Unni 1996)^a^. The Paget's disease study was by Schajowicz et al. (1983)^b^, the radium values were reported by Gössner (1999)^c^, the external irradiation of adults was based on the combined studies of Huvos (1991) and Unni (1996)^d^. Tucker et al. (1987)^e^ assessed the bone cancers developed after the radiotherapy treatment of childhood cancer.

	Osteosarcoma	Chondrosarcoma	Fibrosarcoma/MFH	Ewing's sarcoma	Number of individuals
Spontaneous, all population^a^	41%	25%	8%	12%,	5,676
Spontaneous, 0–20 years old^a^	61%	4%	4%	28%	–
Paget's disease, adults^b^	63%	8%	27%	0%	62
^224^Ra, ^226–228^Ra, mainly adults^c^	59%	7%	30%	0%	92
External radiation, mainly adults^d^	63%	4%	33%	0%	246
External radiation of children^e^	69%	17%	5%	5%	64

There is a low percentage of the spontaneous primary bone tumours occurring in the cortical midshaft of long bones, being about 10% of osteosarcomas, 10% of chondrosarcomas and 14% of malignant fibrous histiocytomas, but these occurence drop to 2–6% for post-irradiation bone sarcomas, including those ^226^Ra-induced (Pool et al. 1983, Huvos 1991, Unni 1996). Spiers and Beddoe (1983) found the frequency of occurrence of spontaneous and radiation-associated osteogenic sarcomas in the long bones of humans to be more associated with the trabecular bone surface area rather than the cortical surfaces.

In addition, Priest et al. (1995) showed that it is rare for osteosarcomas to be induced in animals following intakes of ^226^Ra and ^241^Am, both alpha-emitters that deposit in locations of low bone turnover (cortical or trabecular quiescent bone surfaces). However, intakes of ^224^Ra and ^239^Pu are much more toxic as they preferentially deposit in, and irradiate, sites of high bone turnover, in particular trabecular forming surfaces. In summary, trabecular bone is an important potential bone cancer site, whereas cortical bone is a minor target for radiation bone cancer.

## Niches and oxygen levels of stem cells and progenitor cells at risk

There are two major types of multipotent stem cells found in the skeleton. First, hematopoietic stem cells (HSC) form progenitors of both the myeloid and lymphoid lineages including blood cells, immune cells and osteoclasts that excavate bone. Second, mesenchymal stem cells (MSC) produce bone (osteoblasts), cartilage (chrondroblasts), fat and stromal cells. Many, but probably not all, solid and hematopoietic neoplasms arise from cancer stem cells that are dysfunctional versions of a normal stem cell. These cancer stem cells can self-renew and differentiate into some or all cell types of the tumour (Kelly et al. 2007). The concept of the cancer-stem cell was first developed in leukaemia (AML), but has also been characterised in some solid cancers, including bone tumours that arise from aberrant MSC, e.g., osteosarcoma and chondrosarcoma, (Hope et al. 2004, Gibbs et al. 2005). AML (arising from aberrant HSC), but also CML and ALL have been shown to be maintained by cancer stem cells (Reya et al. 2001). Childhood ALL has been found to be initiated in fetuses by primitive cells of the HSC lineage acquiring characteristic chromosome translocations; these rare mutated cells largely remain covert and quiescent, with only some of them being promoted postnatally to full, overt leukaemia (Greaves et al. 2003). There is still much to learn about radiation's role in the origin of leukemic stem cells, which can involve HSC that became abnormal as a result of accumulated mutations or alternatively, arise via progenitors that regain the stem cell property of self-renewal (Passegué et al. 2003). Normal and cancer stem cells remain quiescent in hypoxic niches, but migrate to or create vascular niches in order to proliferate (Table I). This section provides evidence for partitioning the ICRP's leukaemia and bone cancer targets based primarily on the age-dependent locations of normal and cancer stem/progenitor cells in marrow, and to a lesser extent on the oxygen-dependent radiosensitivity of their niches.

### (1) Leukaemia: Age, cellularity and skeletal targets

Bone marrow is the major site of hematopoiesis in the third trimester fetus, infants, children and throughout adulthood (ICRP 70 1995). HSC are found in medullary cavities in bone, especially in trabecular marrow with high hematopoietic cellularity and little fat. The fetal marrow consists solely of hematopoietic tissue, which is replaced by adipocytes as the individual ages, dropping to an average skeletal cellularity of ∼46% at 70 years old (Richardson and Dubeau 2003). The loss of hematopoiesis in the marrow of long bone cavities is faster in general than in the rest of the skeleton: long bone medullary cavities lack cellularity and hematopoiesis from around 20 years onwards (Cristy 1981).

Acute and chronic leukaemia (Table II) is characterised by unregulated growth of blood cells and their precursors, with acute leukaemia involving relatively immature cells (blasts) compared to the more mature cell types of chronic leukaemia. A CML blast crisis occurs in granulocyte-macrophage progenitor cells that acquire self-renewal capability (Jamieson et al. 2004). CML cases usually are present in a chronic phase before progressing after about three years on average, to an acute form of leukaemia. The initial development of AML appears to involve HSC, while subsequent mutations in progenitors are necessary for full transformation (Hope et al. 2004).

It is generally accepted that HSC and their progenitors are located in two distinct microenvironments or ‘niches’: Firstly, the endosteum or so-called, osteoblastic niche and secondly, the sinusoidal perivascular areas or vascular/endothelial niche (Schofield 1978, Arai and Suda 2007, Lo Celso et al. 2009). Kiel et al. (2005) found that 14% of HSC in murine bone marrow are associated with the endosteum, while most HSC are in contact with sinusoidal endothelia. Experimental evidence generally shows that quiescent HSC (slow cell turnover, with most in the G_0_/G_1_ mitotic cell cycle phase), other primitive hematopoietic progenitor cell types, and AML stem cells, have their highest concentrations close to bone surfaces (Lord 1990, Nagasawa 2006, Colmone et al. 2008, Ishikawa et al. 2007). The 10 μm thick endosteum accommodates small, 3–7 μm diameter, spore-like stem cells (Vacanti 2004) accompanied by 1 μm thin osteoblasts that are bone lining cells. Quiescent HSC may reside in an osteoblastic niche in close contact with Ncadherin^+^ pre-osteoblastic cells and endothelial cells that are MSC progeny, whereas active HSC prefer peri-vascular niches containing mesenchymal reticular cells (Arai and Suda 2007, Kiel et al. 2007).

Less primitive progenitors, pre-leukemic cells and malignant metastasising cells migrate towards the vascular sinusoids located in the central part of marrow in bone cavities (Colmone et al. 2008). Perivascular sites provide nutrients and vascular access to mitotically active cancer stem cells, as well as benefiting HSC/MSC and their differentiated progenitors that replenish the blood system (Sipkins et al. 2005, Kopp et al. 2005). The presence of endosteal and vascular niches supports the proposed partitioning of ICRP's whole marrow target for leukaemia, especially considering that these locations have differing radiosensitivity as described in subsection (3) below.

### (2) Bone cancer: Age, cellularity and skeletal targets

During the third month after conception, a primary then secondary ossification centre is formed in long bones; both ossification centres produce cartilage cavities that fill with marrow. The epiphyseal growth plate produces longitudinal bone growth, while the periosteum provides appositional growth. This skeletal growth is active in the infant, peaks in the adolescent and ends in the young adult: Bone remodelling activity mirrors that of growth but continuing at a low level into old age. In childhood, the spontaneous and radiation-induced bone cancer incidences shows a similar trend to bone growth. The proportions of trabecular bone and cortical bone vary with age, being predominantly trabecular/cancellous bone in the fetus and newborn when skeletal remodelling and proliferative activity is at its maximum (ICRP 70 1995). Cortical bone, which dominates in adults, comprises 80% of the skeletal mass and has a turnover six times slower than trabecular bone. In infants (when cortical bone is scarce), forming surfaces consist of up to 50% of trabecular bone surfaces, while in adults this figure drops to only 3% of cortical bone surfaces and 6% of trabecular bone surfaces.

Human MSC that differentiate into connective tissue cells with osteogenic and chrondrogenic potential are resident in many sites including bone growth plates, the outer cambian layer of the periosteum, and within Haversian canals of cortical bone (Muschler et al. 2003). MSC, originally referred to as colony-forming unit-fibroblasts, support hematopoiesis and osteogenesis, but unlike HSC do not exhibit all stem-cell criteria as they have a more limited capacity for self-renewal and cannot regenerate a whole tissue. Like HSC, MSC seem to occupy two distinct marrow niches (Bianco et al. 2001). Active MSC occupy vascular niches in association with endothelial cells and quiescent MSC are found in close proximity to trabeculae bone surfaces. The number of osteoblastic stem cells, osteoblastic progenitors and osteoblasts in marrow markedly decreases with age after skeletal maturation (Nishida et al. 1999, Muschler et al. 2001). Osteoblast and MSC concentrations, like bone turnover, were observed to be inversely associated to the adiposity of marrow (Moerman et al. 2004). In children, spontaneous and radiation-induced bone cancers are primarily metaphyseal and diaphyseal intramedullary lesions, although benign tumours (e.g., chondroblastoma) are commonly associated with growth plates (Table II). In adults, spontaneous and radiation-induced bone cancers also usually originate in trabecular bone with red marrow, e.g. proximal/distal ends of long bones (Spiers and Beddoe 1983). Trabecular cavity marrow generally exhibits high cellularity and MSC levels, which result in a higher proliferative capacity than cortical bone. The skeletal location of MSC and radiogenic bone cancers support the selection of a radiation target for bone cancer that is associated with remodelling bone found on trabecular bone surfaces.

### (3) Oxygen effect and radioprotection of quiescent stem cells

The preferred microenvironments of differentiated and undifferentiated cells in bone marrow have radically different oxygen tensions and oxygen consumption levels. In a hypoxic environment, stem cells have a low metabolism and a long lifespan. However, stem cells and their shorter-lived progenitors proliferate under higher oxygen pressures. The presence of the hypoxic marker pimonidazole demonstrates that HSC in bone marrow are mostly located in the endosteum at 1% O^2^ or less, i.e., < 1 kPa or 7.6 torr (Parmar et al. 2007). The self-renewal of HSC or MSC, and their maintenance in an undifferentiated state, is enhanced in hypoxic conditions existing close to bone surfaces compared with normoxic conditions found elsewhere in marrow (Grayson et al. 2006). Reactive oxygen species (ROS) arise from exogenous radiation sources, but mostly originate from endogenous mitochondria. The transcription factor, hypoxia-inducible factor-1, (HIF-1) adapts cells to survive the stress of low oxygen conditions (Papandreou et al. 2006). In hypoxic regions, glycolysis is stimulated, oxidative phosphorylation becomes more efficient, and mitochondrial proton leakage and cell death are repressed (Gnaiger et al. 2000). Therefore, hypoxia and the ‘oxygen effect’ protect normal stem cells from ROS, thereby reducing the consequential DNA damage and apoptosis (Moore and Lemischka 2006, Arai and Suda 2007). Similarly, quiescent leukemic stem cells residing in the hypoxic endosteum are resistant to radio-and chemo-therapy and treatment-induced ROS (Ishikawa et al. 2007).

The oxygen enhancement ratio (OER) value is defined as the ratio of the hypoxic and normoxic doses which lead to the same biological effect, such as cell death (Thoday and Read 1949). Radio-sensitivity varies most rapidly with oxygen tensions below about 2% O^2^. The maximum OER value increases with decreasing LET (particle keV lost per μm of track). For example, the OER value utilised by Richardson (2008) for low LET X-rays, was 2.6 in air (21% O^2^). The maximum OER value is 1.3 for high-LET 4.0 MeV alpha particles from ^232^Th decay, compared to a maximum OER of 1.7 for ^224^Ra alpha particles of 5.7 MeV. Therefore, any deleterious radiation effects to MSC, HSC or cancer stem cells residing in the radioprotective microenvironment of the endosteum, compared with radiosensitive vascular niches, will be mitigated by a factor of, at most, two for high LET alpha-radiation and three for low LET beta-, gamma-and X-rays (Table I). The radioresistance of the narrow endosteum will have a minor influence on the overall higher radiosensitivity of the 50 μm thick peripheral marrow that encompasses it.

## The match/mismatch between dosimetry and epidemiological leukaemia incidence

When selecting radiation targets one important aim is for the target dose (Gy) to correspond with the cancer incidence risk obtained from epidemiological studies. This is particularly difficult to achieve for low doses and low dose rates. The choice of target is complicated by factors other than radiation dose that affect the risk of fatal cancer. This section reviews two biological mechanisms that may influence risk: First, cell killing, which has been known for decades to decrease high dose response, and second, recent research on radiation-associated migration of HSC that is hypothesised to be a form of adaptive response.

### (1) Leukaemia risk is reduced by high dose cell killing

Low-LET studies of medical irradiation for Japanese atomic-bomb survivors show a curvilinear dose response for leukaemia, with comparatively lower risk at doses below ∼1 Gy, and cell sterilisation from 3 Gy upwards (Pierce et al. 1996, Little et al. 1999). The leukemic risk and cell killing from this low-LET acute irradiation is compared next with observations from two studies where patients were administered radium and thorium radionuclides and received internal, high dose, high-LET exposures.

German patients, mostly adults, during the 1940s and 50s were treated with multiple injections of bone-seeking radium alpha-emitters for various conditions (Nekolla et al. 2005, Wick and Nekolla 2005). Excess leukaemia was induced when low doses of short-lived ^224^Ra were administered to ankylosing spondylitis patients (Harrison and Muirhead 2003). However, there were lower incidences of leukaemia than expected observed in high dose studies of long-lived ^226^Ra alpha-emitters, including radium dial painters (Spiers et al. 1983). Thorotrast, a thorium dioxide colloid containing ^232^Th and its progeny, was injected mainly into adults as a radiographic contrast medium in the 1930s and 40s. The alpha-radiation exposure of the liver, spleen and bone marrow was long term and probably homogeneously distributed within these organs and tissues. Harrison and Muirhead (2003) compared the risk of radiation-induced leukaemia in patients that received Thorotrast alpha-irradiation with atomic-bomb survivors that were exposed to high-energy gamma rays. When the ICRP value of 20 is employed as the alpha-radiation weighting factor, the hematopoietic malignancies of the Thorotrast exposures were 10 times lower than expected (van Kaick and Wesch 2005). These high alpha-radiation doses sterilised hematopoietic progenitors reducing malignancies (Gössner 2003).

Cardis et al. (1995) conducted the largest international combined study of nuclear industry workers assessing the health risks associated with low dose, mainly low-LET external exposures. The review of these occupational exposures by BEIR VII (2006) concluded that although the risk estimates for leukaemia varied considerably, they were consistent with those on which current radiation protection recommendations are based. Therefore, the current ICRP whole-marrow target for radiation-induced leukaemia provides a reasonable estimate of the haematological cancer risk for low-LET external exposures of adults. Whereas, dosimetric evaluations for high-dose, short-range, high-LET, internal irradiations, based on the same target, appear to overemphasise the risk. This poor assessment of cancer risk is primarily due to cell killing. But could an adaptive response also be playing a role by reducing the biological effects of the internal, chronic irradiation?

### (2) Stem cells migrate after a radiation-insult as an adaptive response?

There is a higher excess relative risk of leukaemia after whole body irradiation of atomic-bomb survivors compared to the risk of leukaemia as a second cancer in patients treated for cervical cancer and uterine cancer with partial-body external beam radiation (Boice et al. 1987, Curtis et al. 1994, Little 2001). Shuryak et al. (2006) contend that the lower leukemogenic risk for partial-irradiation is due to HSC migrating through the blood stream, from niches in bone marrow and spleen that received low doses, to repopulate high dose sites where stem cells have been inactivated. Medical irradiations, often concurrent with chemotherapy, generally involve multiple acute external beam exposures or chronic brachytherapy exposures. It is hypothesised that an early adaptation to the ongoing stress of multiple exposures may reduce the risk of a second cancer.

Real-time imaging has been recently employed to show the localisation of HSC and progenitor cells in medullary cavities of femurs from un-irradiated and irradiated mice. Xie et al. (2009) found HSC randomly distributed in the marrow within 50 μm of the trabecular bone surface of irradiated mice. Controversially, Lo Celso et al. (2009), like some others before them, contend that HSC are normally sparsely localised, if at all, within 30 μm of the endosteum (Kiel et al. 2007). In both studies, other mice were also imaged after receiving around 10 Gy before bone marrow transplantation. HSC were then injected into the irradiated recipients and they localised closer to the endosteal surface than in the unirradiated mice: of the HSC, 47% were now within 15 μm of the endosteal surface according to Lo Celso et al. (2009).

There appears to be good biological reasons to support marrow stem cells being normally located in the vascular niche, but migrating to the endosteal niche in times of physiological stress. A vascularised and well-oxygenated environment provides the essential nutrients for production of progeny and DNA repair by homologous recombination, the latter an energetically demanding but virtually error-free process. Under stress, stem cells that migrate to the hypoxic endosteum will experience the advantages of less free-radical damage due to reduced endogenous and exogenous ROS, as well as increased self-renewal and longevity (Eliasson and Jönsson 2010). Ionising radiation, especially alpha-particles, produce comparatively more DNA double strand breaks than mitochondrial proton leakage. A downside of hypoxia is slow HSC turnover (∼1 year in nonhuman primates) and reduced DNA repair (Mahmud et al. 2001). This leads to a greater reliance on double-strand breaks being repaired by error-prone replication-independent processes (Rodríguez-Jiménez et al. 2008). No matter what, the long-term stress associated with ageing will result in stem cells, even if quiescent, accumulating mutations and exhibiting shorter telomeres, as well as accelerated stem cell exhaustion and senescence (Nijnik et al. 2007; Richardson 2009b).

Several biological mechanisms – including DNA-repair processes, apoptosis, and the immune response – have been expounded in the literature to explain the adaptive response of somatic cells to stress, such as heat or radiation (Streffer 2009). In many situations a protective adaptive response by radiation is demonstrated by a low acute dose of 5– 200 mGy, given 4–24 h before a higher challenging dose of 1 to several Gy. The mice in the experiments of Lo Celso et al. (2009) and Xie et al. (2009) were irradiated at higher levels than for adaptive response experiments in general. After Xie et al. (2009) exposed their mice to 10 Gy, and in response to the resulting bone marrow damage, HSC migrated into the radioprotective endosteum, which they then occupied for > 10 months. It would be of interest to learn if this migratory behaviour of stem cells from a vascular niche to a hypoxic one occurs for shorter times at the lower doses usually employed to demonstrate adaptive response; and in addition, to also determine if this adaptation is responsible for reducing the late effects of multiple or chronic radiation doses compared with acute exposure.

## Biological mechanisms linking radiogenic fibrosis and marrow cancers

Although the targets proposed were selected to allow a prediction of excess cancers attributable to the radiation dose, there are other factors besides dose that influences the risk. This section reviews the evidence that fibrosis and pre-existing lesions are associated with aging and radiation-induced bone cancer. Conversely, it is suggested that where population groups display a rarity of these lesions, as for all children and Asians adults, this will modify the risk of this lesion-associated, late-effect neoplastic development. Therefore, the bone cancer targets of children and adults will differ as different factors such as pre-existing lesions and growth influence their risk. Lastly, this section reviews the recently published research on biological mechanisms which may suggest a link between radiation, inflammation, fibrosis and leukaemia.

### (1) Pre-existing lesions, fibrosis and radiation-induced bone cancer in adults

Several researchers have observed that following a radium intake a mainly acellular, fibrotic layer forms on bone surfaces. Lloyd and Henning (1983) contended that this 8–50 μm thick microenvironment found in the neighbourhood of a bone tumour predisposed the site to radiation-induced bone cancers. Gössner (1999) hypothesised that preexisting lesions (e.g., Paget's disease, bone infarct, fibrous dysplasia), or radiation-induced bone lesions (e.g., radiation osteitis), and associated fibrosis might be critical for the induction of bone sarcomas of fibroblastic and fibrohistiocytic origin. The case is made in the next subsection that this hypothesis is more relevant to adults than children. Gössner's observation was made on the evidence that adults receiving radium or external beam radiotherapy showed the same spectrum of bone tumour types as adults with pre-existing bone lesions (rows 4, 5, 6; Table V). For example, in patients with Paget's disease Schajowicz et al. (1983) reported a high fraction (27%) of fibrosarcomas and malignant fibrous histiocytomas (row 4, Table V). In a population comprising of mainly adults, fibrosarcoma and malignant fibrous histiocytoma are about three times more prevalent as a radiation-induced tumour than as a spontaneous tumour (rows 2, 5, 6; Table V).

Similar to radiation-induced cataracts, alpha particle irradiation of the skeleton appears to have a deterministic effect by inducing premature aging, probably by increased inflammation and fibrosis, the depletion of stem cells in marrow, and increased mutations of the remainder stem cells (Richardson 2009b). The average age of female US radium-dial painters at first exposure was just 20 ± 5 years old (Rowland et al. 1983). The appearance time of the ^226,228^Ra-induced bone sarcomas was long, on average 27 ± 14 years. The latent period was shortened dependent on the average skeletal dose. With the characteristics of a dose threshold, at lower doses the aging effect in these young women was small and inconsequential, as the cancer induction period was likely greater than the human life span (Evans et al. 1969).

Unlike leukaemia, bone sarcomas are rarely induced below skeletal doses less than 10 Gy (Brenner et al. 2003). This applies to adult radium-dial painters (BEIR IV 1988), Mayak workers exposed to plutonium (Shilnikova et al. 2003), ankylosing spondylitis patients treated with ^224^Ra (Wick and Nekolla 2005), and children receiving radiotherapy (Tucker et al. 1987). The high dose threshold for radiogenic bone cancer may be related to the finding that at low or moderate doses most tissues/organs studied express more fibrosis cytokine than bone marrow (Chang et al. 1995, Martin et al. 2000): the possible involvement of this biological mechanism is explored further in subsection (4) below. In light of the association of radiogenic bone cancer and fibrosis, Lloyd and Henning (1983), supported by Gössner (2003), proposed increasing the thickness of the ICRP radiation target from the endosteum of 10 μm thickness to a 50 μm marrow layer adjacent to the bone surface, where fibrosis associated with spontaneous or radiation-induced bone lesions is commonly located.

### (2) Higher bone cancer rates in children are not associated with pre-existing lesions

Children and adults receiving high medicinal doses of ^224^Ra had a statistically significant increase in bone cancer risk with decreasing age at exposure (Nekolla et al. 2005). In patients treated for tuberculosis and other diseases by an injectant containing ^224^Ra, the incidence of bone sarcoma was about 14 times higher in the 1-to 5-year-olds, compared to adults > 20 years old (Spiess 1969). Children receiving radiotherapy had an increased risk of bone sarcomas above doses of 10 Gy (Tucker et al. 1987). The mean period from the treated first cancer to the secondary bone cancer was around 10 years, a shorter latency than for adults receiving similar therapy.

Pre-existing lesions of bone are more common in adults, whereas benign tumours are more common in children. Around half of spontaneous bone tumours in American children are non-malignant (Unni 1996), although the transformation from benign to malignant form is rare. Children receiving radiotherapy developed malignant bone cancers of a similar type to those arising in a population spontaneously, but with a lower incidence of Ewing's sarcoma (rows 3, 7; Table V). Irradiated children developed fewer bone sarcomas of fibroblastic and fibrohistiocytic origins (Tucker et al. 1987); these sarcomas are relatively common among the radiation-induced bone cancers of adults (see previous subsection). The ICRP justifies the employment of a peripheral marrow target on the basis that radiogenic cancers are associated with fibrosis or pre-existing lesions. Although, Gössner's (1999) observation is important for adults, it has little relevance to irradiated infants and children for whom pre-existing lesions and fibrosarcoma/malignant fibrous histiocytoma are rare. Instead, rapid skeletal remodelling and growth are probably the dominant factors linked with both spontaneous and radiogenic bone cancers of children and adolescents, thereby supporting the partitioning (and perhaps age-dependent weighting) of the peripheral marrow target into that associated with quiescent and forming bone surfaces.

### (3) Rarity of Paget's disease and lack of bone cancer in Japanese atomic-bomb survivors

Paget's disease of bone has a strong genetic component (Albagha et al. 2010) and is very common in some elderly Caucasian populations in Europe and US; it is less common in Scandanavia and extremely rare in Asia and Africa (Hashimoto et al. 2006). Paget's disease is characterised, similar to radiation osteitis, by abnormal and rapid osseous remodelling. It is rarely found in adults under 45 years old and becomes more common with ageing (Cooper et al. 2006). The disease is a benign condition with the potential for malignant transformation. Those with Paget's disease have an approximately 10-fold higher risk than the general population in developing bone sarcomas (Wick et al. 1981, Ghadirian et al. 2001). In most countries, Paget's disease is estimated to occur in 0.1–5% of individuals. However, in Japan, spontaneous bone cancer rates, including those in childhood, are relatively low when compared to international values (Parkin et al. 1993). In particular, there are low incidences of osteosarcoma in Japanese and Chinese adults aged over 50 years, which could be related to the lack of Paget's disease (Guo et al. 1999).

While a significant increase in bone-cancer mortality has been seen in adult radium dial painters in Europe and the US, bone cancer is among the few exceptions to the full spectrum of spontaneous cancers reported in excess among adult atomic-bomb survivors exposed to relatively low doses (Brenner et al. 2003, Preston et al. 2007, Richardson 2009b). It is acknowledged that the characteristics of these two radiation exposures were very different in terms of homogeneity and duration of the exposures, the mean dose, LET and relative biological effectiveness (RBE). Nevertheless, Little (2001) found a strong increasing trend of bone cancers with atomic-bomb dose for those exposed as children (excess relative risk, 16.5), even though there was no significant association for adult-exposed survivors (excess relative risk, −0.3).

One reason for the relative lack of excess bone cancers in atomic-bomb survivors could be due to only 323 of the 86,661 exposed survivors having whole body (life-threatening) doses over 4 Gy (Pierce et al. 2008) and possibly none exceeding a 10 Gy skeletal dose threshold. Even if radiation can induce bone cancers at doses < 10 Gy, a second factor may be the extremely low prevalence of Paget's disease in Japan, estimated at just 2.8 cases per million capita (Hashimoto et al. 2006). Follow-up studies of patients administered alpha-emitting Thorotrast (Mori et al. 2005, van Kaick and Wesch 2005) have exhibited excess bone cancers at significantly higher incidences (*p* < 0.05) in at least four of the six countries studied. Further analysis may provide the opportunity to statistically determine if irradiated adults from countries where Paget's disease of bone is very common (i.e., Germany, Portugal and the US) have a higher predilection to develop excess bone sarcomas than citizens from countries where Paget's disease is uncommon or extremely rare (i.e., Denmark, Sweden and Japan). Therefore, it appears the ICRP's present bone cancer target, the peripheral marrow adjacent to quiescent surfaces, has limited relevance to Asian and Nordic adults, and children globally, for whom pre-existing bone lesions are very rare.

### (4) Involvement of fibrosis and the wound model in radiation-induced marrow neoplasms

One could speculate from recent research that radiation inflammation, observed as a low to high dose-dependent effect in atomic-bomb survivors (Hayashi et al. 2008), and associated fibrosis, may be important factors not only for radiogenic bone cancers but also in the induction of leukamia. The fat cell content of adult marrow increases with aging and irradiation, and probably contributes to increased inflammation due to hormonal effects of the ‘fat hormone’ leptin (Iikuni et al. 2008). Myelodysplastic syndrome (MDS), sometimes called preleukaemia, is a clonal disorder of HSC (Kuramoto et al. 2002). MDS is also associated with both aged and irradiated marrow. MDS patients present more angiogenesis in bone marrow than normal (Medinger and Mross 2010). The resulting increase in radiosensitivity may be a factor in the MDS excess relative risk being ∼13 Gy^−1^ in atomic-bomb survivors, which is much higher than the risk of leukaemia, 3.8 Gy^−1^ and all other cancers, 0.3 Gy^−1^ (Pierce et al. 1996, Tsukasaki et al. 2007). Higher occurrences of fibrosis and MDS are also prominent in Thorotrast administered to patients (Visfeldt and Andersson 1995, Ishikawa et al. 2001). The ineffective hematopoiesis and marked reticulin fibrosis displayed by MDS and caused by high radiation doses or chemotherapy can lead to AML/erythroleukaemia or marrow failure. As shown earlier (Table III), the AML incidence as a percentage of the total leukaemias is enhanced in atomic-bomb survivors (2-fold) and Thorotrast patients (3-fold) compared with the spontaneous incidence rate. A quarter of the AML cases were erythroleukaemias in Danish Thorotrast patients, whereas only a few percent occur spontaneously (Visfeldt and Andersson 1995).

Transforming growth factor β1 (TGF-β1) is elevated in plasma during radiotherapy and involved in the proliferation of MSC, which lead to reticulin and collagen fibrosis in bone marrow by fibroblasts (Martin et al. 2000). TGF-β1 is a cytokine that regulates defence response to injury. Latent TGF-β1 is activated by ROS from radiation and other sources/stresses such as chemicals or heat (Jobling et al. 2006). Progenitor cells from patients with MDS-derived AML display a mixed response to TGF-β, with AML cells in only four of seven cases escaping TGF-β mediated growth inhibition (Koschmieder et al. 2001). The involvement of TGF-β1 is more transparent in transforming pre-leukeamic cells with the TEL-AML1 chimeric fusion gene into childhood ALL (Ford et al. 2009). The biological mechanism transforming MDS to AML/erythroleukaemia is complex and as yet unclear. However, recent research suggests that radiation's ability to produce inflammation and fibrosis may be important factors in the induction of not only bone cancer but also leukaemia.

## Discussion

The former ICRP target, the 10 μm thin endosteum may be a questionable size for a target as a measure of radiation dose and risk, because of the genetic instability observed in bystander non-irradiated marrow stem cells and progeny (Kadhim et al. 2004): this could be due to a radiation-induced inflammatory response (Lorimore et al. 2005). Cell-to-cell signalling regulates, in part, cell growth, differentiation and apoptosis. It is conceivable that radiation has an influence on the niche cells that regulate stem cell fates by affecting signaling molecules, the extracellular matrix, and cell adhesion molecules (Arai and Suda 2007). Therefore, irradiating support cells of stem-or progenitor-cells may provoke long-range detrimental biological effects due to disruption of the support cell's communication.

To be accurate, it is only the haematopoitic tissue which constitutes a leukaemia target rather than whole red bone marrow that ICRP (ICRP 23 1975) describes as consisting of 40% fat cells in addition to hematopoietic tissue in adults. The age-dependent alpha-particle dosimetry of hematopoietic marrow, rather than red marrow, was first carried out employing analytical methods (Richardson et al. 1991), whereas more sophisticated Monte Carlo simulations enable alpha-and beta-radiation dosimetric analyses of the skeleton, both in the quiescent (Eckerman and Stabin 2000, Richardson and Du-beau 2003, Watchman et al. 2005) and remodelling states (Richardson et al. 2007, Nie and Richardson 2009). For a minority of radionuclides and compounds, the dose to hematopoietic marrow and adipocytes can be markedly different and at variance with the average dose to homogeneous red marrow. This dose variation can occur for short-range radiations and radionuclides of elements or compounds that have higher activities in either adipocytes e.g. carbon, radon (high gaseous solubility), or alternatively, higher activities in hematopoietic tissue, such as plutonium (Leggett 1985) as well as the lean tissue seekers, potassium and polonium. A representative dose for the whole skeleton by short range irradiation of bone cancer and leukemic targets can be obtained by summing dose to these targets in various bones, weighted by a factor involving the trabecular or cortical bone's cellularity, an indicator of proliferative capacity (Richardson et al. 1991). If this weighting process is applied to the proposed ICRP target, the cortical medullary endosteum, in essence, has virtually no influence on the radiation dose to the bone cancer target at any age.

Increasing the peripheral marrow layer from 10 μm to 50 μm will affect the alpha particle dose to the bone cancer target from activity located in trabecular bone or marrow (Nie and Richardson 2009). This is due to the limited maximum range of alpha particles, e.g., 10 μm in soft tissue for ^144^Nd, 1.8 MeV particles and 90 μm for ^212^Po, 8.8 MeV. The same is true for low energy, shorter-range beta-emitters, such as ^14^C with a mean range of 40 μm in water. Alpha-particle activity was modeled by Nie and Richardson (2009) as a uniform source in bone marrow, bone volume and on bone surface. The ICRP's decision to move to a 50 μm marrow layer results in a relatively small increase in dose to the bone cancer target from alpha-emitters deposited in marrow, e.g., Thorotrast. Conversely, a relatively large decrease in target dose results from alpha-emitters homogeneously deposited in bone volume, and particularly for alpha-emitters on bone surface. The ratio comparing the 50 μm peripheral marrow dose to the 10 μm endosteum dose is around 1:3.4 for ^226^Ra, ^224^Ra and ^239^Pu on bone surface, and 1:4.3 for ^232^Th. The ratio of the doses is 1:5 for alpha-or beta-particles with a range equal to or less than the width of the endosteum, e.g., ^144^Nd. Therefore, by employing the 50-μm target over the 10 μm one, the ICRP will effectively reduce by as much as 5-fold the alpha-particle target dose from bone-volume and bone-surface seekers, which in turn lessens the predicted risk of bone cancer (and conversely, increases the RBE more in line with ICRP recommendations).

Fractional weighting of the endosteum and central marrow regions could be chosen based on the calculated leukemic target dose and risk, and compared with the corresponding excess leukaemia observed in epidemiological studies. A 10 μm thick endosteum takes up only about 4% of the trabecular marrow cavity volume of an adult; however this rises to ∼9% for the newborn. These values are derived assuming the dimensions and morphology of a “spherical” trabecular cavity, as evaluated by Richardson and Dubeau (2003). The proposed ICRP target of average dose to trabecular red bone marrow in effect weighs the dose to the endosteum and central marrow regions by a ratio of about 1:24 and 1:10 for adults and newborn, respectively. However, taking account of the radioprotection of the hypoxic endosteum and a maximum OER of 3 and 2 for low-and high-LET irradiation, the 10-mm endosteum to central marrow dose ratio would be weighted by about 1:48 and 1:20 for adults and newborn exposed to high-LET radiation. This places less emphasis on the dose to the endosteum and for bone-seeking alpha-emitters, e.g., ^226^Ra, would lessen the overall dose to the leukemic target and go some way, besides cell killing, to explaining the virtual lack of excess leukaemias in radium studies (Spiers et al. 1983, BEIR IV 1988). It is worth noting that modified weighting of dual non-over-lapping, hematopoietic targets does not greatly change the leukemic dose and risk assessment for homogeneous irradiation of whole marrow by external or internal sources, e.g., atomic-bomb gamma rays or Thorotrast.

The use of the marrow targets proposed here will require considerable changes to dosimetric methods presently employed by ICRP to ascertain radiation dose and risk. Firstly, new dosimetric methods are needed to assess the radiation dose to the peripheral marrow associated with both the quiescent bone surface and bone remodelling. Nie and Richardson (2009) conducted a dynamic simulation that studies the temporal changes of short-range radionuclides incorporated in a trabecular bone remodelling compartment. This compartment consists of a well-oxygenated vascular microenvironment located within a canopy of bone-lining cells. Angiogenesis, and consequently the radiosensitivity of marrow, is enhanced in the vicinity of bone remodelling. The stem/progenitor cells participating in bone remodelling are in close proximity to radioactivity incorporated in new bone. Secondly, biokinetic models provide the temporal variation in radioactivity from an internal exposure. The models currently employed by the ICRP do not have compartments specifically assigned to marrow targets associated with bone-remodelling. A physiological skeletal biokinetic model has been developed that differentiates between quiescent and forming bone surfaces (Richardson, 2010). This modification is essential for meaningful risk assessment of marrow neoplasms arising from irradiating infants and children.

The development of cancer is a multistage process; animal studies have shown radiation to induce more initiation than promotion events (BEIR VII 2006). It is well known that radiation has an enhanced ability, compared to mitochondrial-generated ROS, to cause DNA double-strand breaks and presumably, cancer-initiating chromosome translocations. The evidence points to obstetric X-rays initiating childhood leukaemia *in utero* with no dose threshold (Bithell and Stewart 1975). But there again, radiation-induced inflammation and fibrosis appears to have a mainly promotional role in adult bone cancers. Pre-existing lesions play a similar role. Paget's disease of bone is present in around 2% of individuals of European ancestry and has an age-threshold of ∼45 years. The average skeletal dose threshold of about 10 Gy evident for the induction of bone tumours in radium dial painters (Evans et al. 1969) may be partly due to premature aging, advancing Paget's disease and the shortening of the cancer latent period in these young women of mainly European descent. However, even this strong case for a dose threshold for bone cancer incidence after ingestion of radium by dial painters is disputed by Leenhouts and Brugmans (2000) who employ a two-mutation carcinogenesis model that supports a linear-quadratic dose-effect relationship with lowered risk at low dose.

## Conclusion

Partitioning the ICRP dosimetry targets of trabecular whole bone marrow (into central marrow and endosteum) and peripheral marrow (into quiescent and forming bone surfaces) will improve the risk assessments for radiation-induced leukaemia and primary bone cancer, especially for children. A summary of the main points:

- Rarity in Japanese of Paget's disease of bone, characterised by hypervascular marrow and focal increases in bone turnover, is a factor in the lack of excess bone cancers in adult atomic-bomb survivors.
- Pre-existing lesions are not an important pre-requisite for radiogenic bone cancer in children whereas growth is, therefore supporting the additional target of peripheral marrow adjacent to forming bone surfaces.
- Quiescent, normal stem cells and cancer-initiating cells reside in a hypoxic niche located in the endosteum layer and have approximately a 2- to 3-fold greater degree of radioprotection com pared to vascular niches.
- HSC and MSC migrate to microvessels to repopulate the blood cells and remodel bone: Cancer stem cells similarly occupy or create vascular niches to produce malignant progeny and metastasise. Morphological and epidemiological evidence, as well as variable niche radio-sensitivity, supports the need to partition the ICRP's whole marrow and peripheral marrow targets.
- It is suggested that stem cells in central marrow migrate to the hypoxic endosteum after a radiation exposure as an adaptive response, thereby showing temporal variation in target location.
- Radiation-induced inflammation, fibrosis and consequent progression in pathogenesis from a non-malignant to malignant form support a ‘radiation wound to malignancy’ model for bone cancer and perhaps leukaemia.
- To assess the risk of bone cancer, especially in children, requires new biokinetic compartmental models to calculate the radiation dose associated with forming bone surfaces and growth plates.
- There are indications that radiation at low doses can induce chromosome translocations and at higher doses accelerate aging by inflammation, shorten the cancer latent period, and advance fibrosis/MDS and stem cell senescence.

The aim of this paper is to propose radiation dose targets, based on current knowledge, for assessing the risk of inducing marrow neoplasms, especially for the third trimester fetus, infants and children. However, there are many uncertainties, such as the exact origin of cancer stem cells and radiation's contribution to the carcinogenic process, especially at low doses. Therefore, the proposed hematological targets have a speculative element until there is better clarification of radiation's role in the cancer stem cell process which may well differ for childhood and adult cancers. Consequently, like the ICRP targets, the targets proposed here will need future modification as biological and epidemiological knowledge advances.
